# Regulatory role of non-coding RNA in ginseng rusty root symptom tissue

**DOI:** 10.1038/s41598-021-88709-3

**Published:** 2021-04-28

**Authors:** Xingbo Bian, Pengcheng Yu, Ling Dong, Yan Zhao, He Yang, Yongzhong Han, Lianxue Zhang

**Affiliations:** 1 State Local Joint Engineering Research Center for Ginseng Breeding and Development, Jilin Agricultural University, Changchun, China; 2 College of Chinese Medicinal Materials, Jilin Agricultural University, ChangchunJilin, 130118 China; 3 Jilin Provincial Ginseng and Pilose Antler Office, Changchun, China

**Keywords:** Plant sciences, Bioinformatics

## Abstract

Ginseng rusty root symptom (GRS) is one of the primary diseases of ginseng. It leads to a severe decline in the quality of ginseng and significantly affects the ginseng industry. The regulatory mechanism of non-coding RNA (ncRNA) remains unclear in the course of disease. This study explored the long ncRNAs (lncRNAs), circular RNAs (circRNAs), and microRNAs (miRNAs) in GRS tissues and healthy ginseng (HG) tissues and performed functional enrichment analysis of the screened differentially expressed ncRNAs. Considering the predictive and regulatory effects of ncRNAs on mRNAs, we integrated ncRNA and mRNA data to analyze and construct relevant regulatory networks. A total of 17,645 lncRNAs, 245 circRNAs, and 299 miRNAs were obtained from HG and GRS samples, and the obtained ncRNAs were characterized, including the classification of lncRNAs, length and distribution of circRNA, and the length and family affiliations of miRNAs. In the analysis of differentially expressed ncRNA target genes, we found that lncRNAs may be involved in the homeostatic process of ginseng tissues and that lncRNAs, circRNAs, and miRNAs are involved in fatty acid-related regulation, suggesting that alterations in fatty acid-related pathways may play a key role in GRS. Besides, differentially expressed ncRNAs play an essential role in regulating transcriptional translation processes, primary metabolism such as starch and sucrose, and secondary metabolism such as alkaloids in ginseng tissues. Finally, we integrated the correlations between ncRNAs and mRNAs, constructed corresponding interaction networks, and identified ncRNAs that may play critical roles in GRS. These results provide a basis for revealing GRS's molecular mechanism and enrich our understanding of ncRNAs in ginseng.

## Introduction

Ginseng (*Panax ginseng* Mayer) is a significant medicinal material with high nutritional value and medicinal value^1^. Due to the limitations of planting areas and planting patterns, it is more important to improve the yield and quality of ginseng for the ginseng industry's sustainable development^2^. As we all know, ginseng has higher requirements on the growth environment and longer growth life, so it is easy to occur in the growth process of various diseases, affecting its yield and quality^3^.

Ginseng rusty root symptom (GRS) is one of the most common diseases in ginseng cultivation and production. It produces reddish-brown spots on the periderm of ginseng roots, and with the increase of planting years, the spots may gradually expand, which will lead to a decline in commodity-grade and ginseng quality. Previous studies have found that chitosan application induces rusty root symptoms, and a variety of phenolic compounds and elements accumulate in rusty root tissues^4^. In particular, Al and Fe's accumulation may promote the accumulation of phenolic compounds and the activation of enzymes related to their oxidation. The activity of various antioxidant substances and antioxidant enzymes in rusty root tissues is significantly increased, preventing phenolic compounds from being oxidized^5^. The microorganisms produce pectinase, cellulase, and ligninase that damage the cell walls of ginseng roots, causing rusty root symptoms exacerbated by the application of Fe^3+^^6^. The presence of potential pathogenic fungi *Ilyonectria* has been reported, and non-biological factors such as Al, Fe stress in the rusty root formation process seem to be closely related^7–12^. However, the molecular regulatory mechanisms of GRS are still elusive.

In recent years, increasing studies have used the Illumina RNA-seq platform based on transcriptome analysis to explore plant responses to abiotic or biological stresses and understand their associated molecular mechanisms. In organisms, there is usually an ncRNA, in addition to mRNA, that does not encode a protein but has an important regulatory function. According to their size, ncRNAs can be subdivided into small ncRNAs (< 200 nucleotides long), including miRNAs and lncRNAs with a length > 200 bp and circRNAs, consisting of a continuous closed loop^13–15^. It has been shown that ncRNA is closely related to plant resistance and homeostasis regulation^16,17^. Although ncRNAs in ginseng have been studied, the role of ncRNAs in GRS has not been reported^18–20^. Besides, with the deepening of the research on ncRNA's function, the regulatory mechanism between ncRNA and genes has been gradually improved. The construction of a gene regulatory network has become an essential strategy to reveal the regulatory mechanism of diseases and important plant characters^21,22^.

In the present study, we identified ncRNAs in GRS tissue, and HG (healthy ginseng) tissue, the characteristics of the identified ncRNA were also described. The differentially expressed ncRNAs were screened, and their target genes were predicted. And the GO (Gene Ontology) annotation was used for classification and functional analysis of target genes, and the biological pathways of these genes were revealed through KEGG (Kyoto Encyclopedia of Genes and Genomes) (www.kegg.jp/kegg/kegg1.html) pathway analysis^23^. Finally, we integrated the correlation between ncRNA and mRNA to construct the interaction network.

## Results

### Quality control and de novo assembly of RNA-seq reads

We mainly analyzed lncRNA and circRNA obtained from the cDNA libraries. The sequencing of each ginseng sample produced over 100 million reads. After filtering adaptors and low-quality reads, we got high-quality clean reads, accounting for more than 96% of the raw reads (Table S1). In the sequencing results of six sRNA libraries, each sample produced more than 12 million reads. Similarly, by removing Adapter related reads and low-quality reads, we get high-quality clean reads, accounting for more than 96% of the original reads (Table S2).

### Characteristics of ncRNAs

#### Identification results and characteristics of lncRNAs

**Figure 1:**
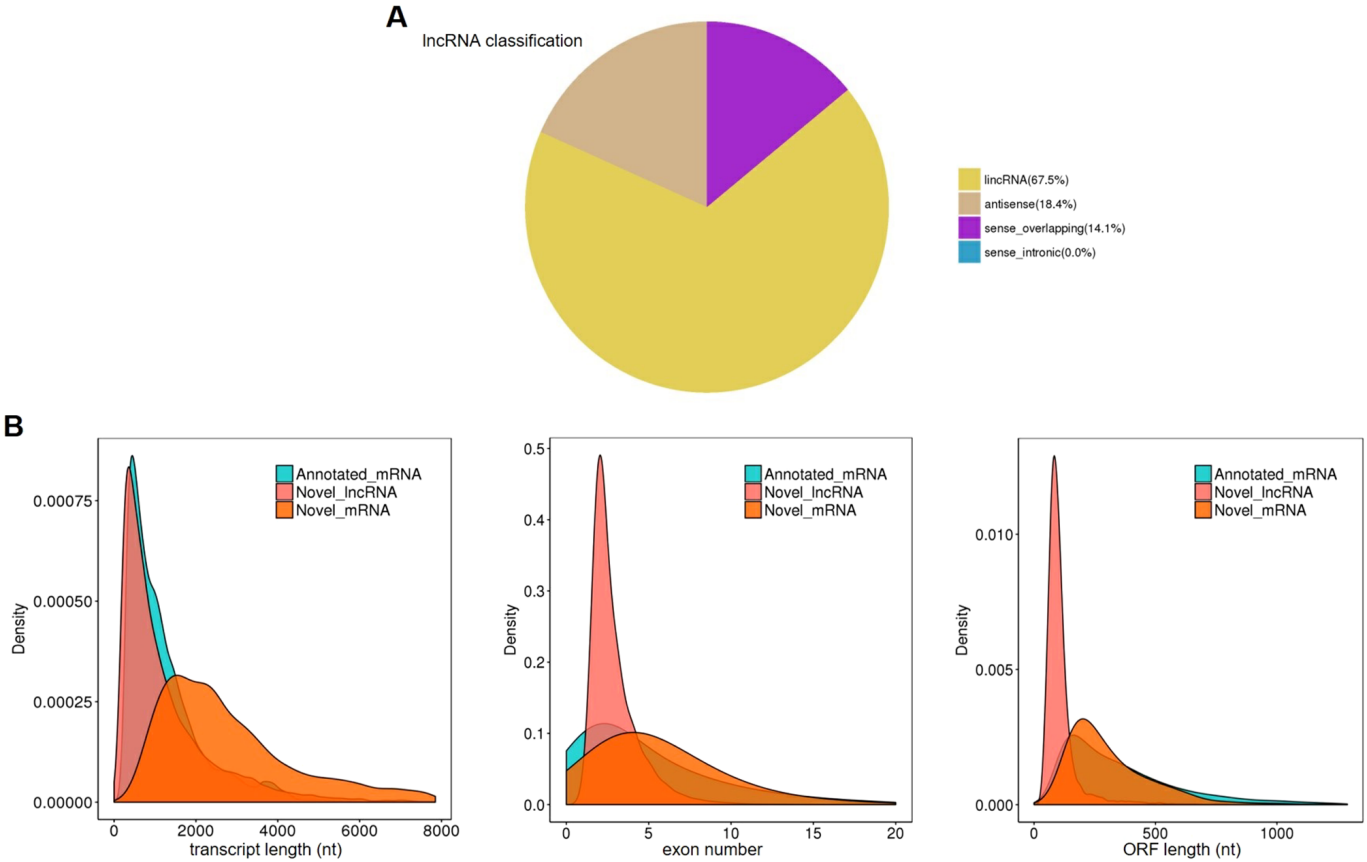
Prediction results and characteristics of lncRNAs. (**A**) lncRNAs classification; (**B**) comparison of length, number of exons and open reading frame between novel lncRNAs and mRNAs. Image generated with R 3.6.3 (https://www.R-project.org)^51^ ggplot2 package (https://ggplot2.tidyverse.org).

By comparison with transcript databases and screening for length and coding potential, we predicted a total of 17,645 lncRNAs. The complete lncRNAs prediction results are shown in dataset 1, and the sequence information are freely available in the NCBI database under accession no. PRJNA713913. As shown in Fig. 1A, 11,914 (67.5%) of all lncRNAs were lncRNAs located in intergenic regions (lincRNAs), 3241 (18.4%) antisense lncRNAs, and 2490 (14.1%) sense lncRNAs. We compared the newly predicted lncRNAs with mRNAs, and most of the lncRNAs were less than 2000nt long, which is consistent with previous research^18^. Besides, less exon and shorter Open Reading Frame (ORF), which confirms that our predicted lncRNAs are more in line with the general characteristics of lncRNAs (Fig. 1B).

#### Identification results and characteristics of circRNAs

**Figure 2:**
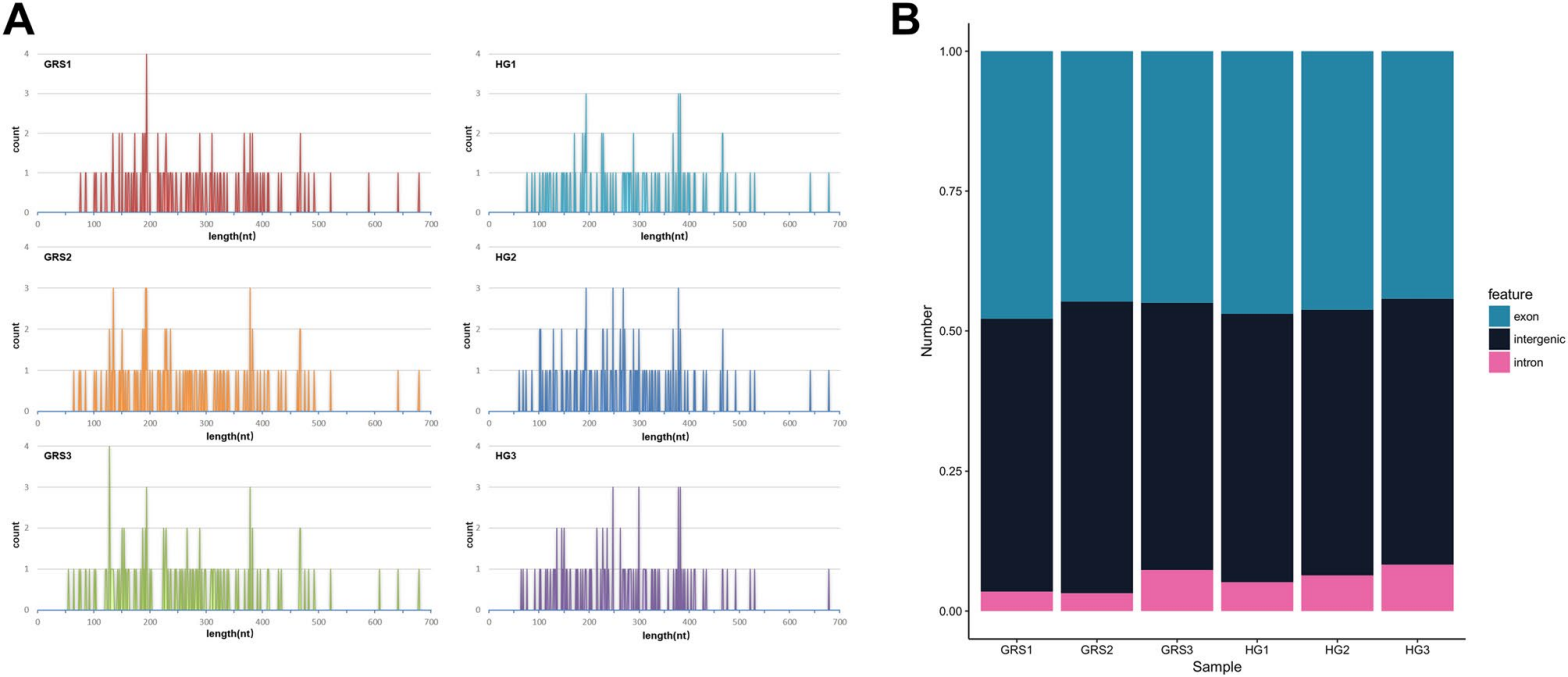
Identification results and characteristics of circRNA. (**A**) Length distribution of circRNAs; (**B**) circRNAs classification. Image generated with Microsoft Office Excel and R 3.6.3 (https://www.R-project.org)^51^ ggplot2 package (https://ggplot2.tidyverse.org).

By identification, we obtained a total of 245 circRNAs in six samples. The complete lncRNAs prediction results are shown in dataset 2, and the sequence information are freely available in the NCBI database under accession no. PRJNA715297. The length distribution is shown in Fig. 2A, and the identified circRNAs ranged from 50 to 700 nt, and the majority of circRNAs were less than 500nt in length. Based on sequence composition, these circRNAs can be divided into exon, intergenic, and intron components (Fig. 2B).

#### Identification results and characteristics of miRNAs

The complete miRNAs prediction results are shown in dataset 3, and the sequence information are freely available in the NCBI database under accession no. PRJNA715301. A total of 299 miRNAs were identified, of which 225 were novel miRNAs. As shown in Table 1, we performed statistics on the comparison and annotation of all sRNA with rRNA, tRNA, snRNA, and snoRNA, as well as repeat. The proportion of rRNA in each sample ranged from 12.32 to 29.92%, indicating good sample quality. The highest proportion of known miRNA in all samples was 2.64%, while the highest proportion of novel miRNA was 0.84%.

**Table 1:** Statistical overview of various sRNA types.

Types	GRS1 (percent)	GRS2 (percent)	GRS3 (percent)	HG1 (percent)	HG2 (percent)	HG3 (percent)
Known miRNA	73,679 (1.79%)	145,816 (1.39%)	120,485 (1.19%)	241,032 (2.64%)	140,930 (2.06%)	226,231 (2.20%)
rRNA	825,401 (20.03%)	1,292,397 (12.32%)	1,501,552 (14.86%)	1,783,348 (19.54%)	2,049,090 (29.92%)	3,258,171 (31.63%)
tRNA	180,683 (4.38%)	997,735 (9.51%)	1,643,876 (16.27%)	408,644 (4.48%)	166,521 (2.43%)	373,450 (3.63%)
snRNA	2364 (0.06%)	5193 (0.05%)	4094 (0.04%)	3337 (0.04%)	2629 (0.04%)	3539 (0.03%)
snoRNA	3235 (0.08%)	5761 (0.05%)	5117 (0.05%)	3973 (0.04%)	3696 (0.05%)	4990 (0.05%)
Repeat	443,558 (10.76%)	1,270,460 (12.11%)	1,005,613 (9.95%)	1,028,449 (11.27%)	651,718 (9.52%)	946,467 (9.19%)
Novel miRNA	34,729 (0.84%)	74,360 (0.71%)	58,377 (0.58%)	68,678 (0.75%)	55,896 (0.82%)	79,584 (0.77%)
Other	2,556,886 (62.06%)	6,697,710 (63.86%)	5,762,731 (57.06%)	5,587,575 (61.24%)	3,778,625 (55.16%)	5,407,542 (52.50%)

**Figure 3:**
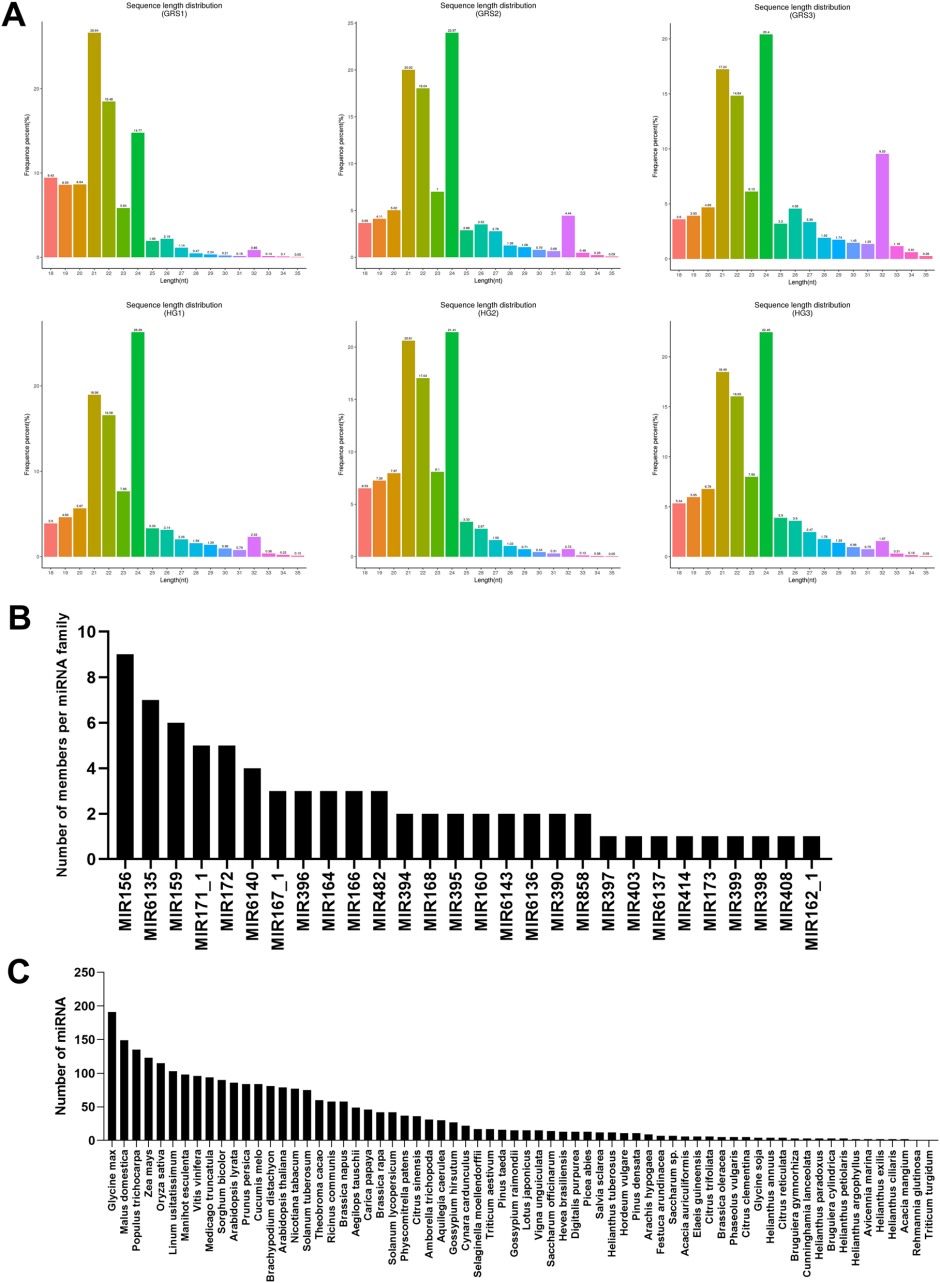
Identification results and characteristics of miRNA. (**A**) Statistics of length distribution of sRNA fragments obtained; (**B**) miRNA family statistics of miRNAs in ginseng tissue; (**C**) statistics of miRNA in ginseng tissue in other species. Image generated with R 3.6.3 (https://www.R-project.org)^51^ ggplot2 package (https://ggplot2.tidyverse.org).

The sRNA length distribution obtained by screening is shown in Fig. 3A, and the sRNA length distribution of all six samples was mainly concentrated between 21 and 24 nt. Besides, we performed a family analysis of the obtained known miRNAs and new miRNAs to count their miRNA family affiliations. As shown in Fig. 3B, a total of 76 miRNAs belonged to 28 miRNA families. Among these miRNA families, MIR156, MIR6135, and MIR159 are the larger three families, containing nine, seven, and six miRNAs. Considering that miRNAs are highly evolutionarily conserved, we counted the miRNAs identified in ginseng tissues in other species. As shown in Fig. 3C, many of the miRNAs identified in the ginseng tissues have high homology with *Glycine max*, *Malus domestica*, *Populus trichocarpa*, *Zea mays*, and other species.

### Differential expression analysis of ncRNAs

**Figure 4:**
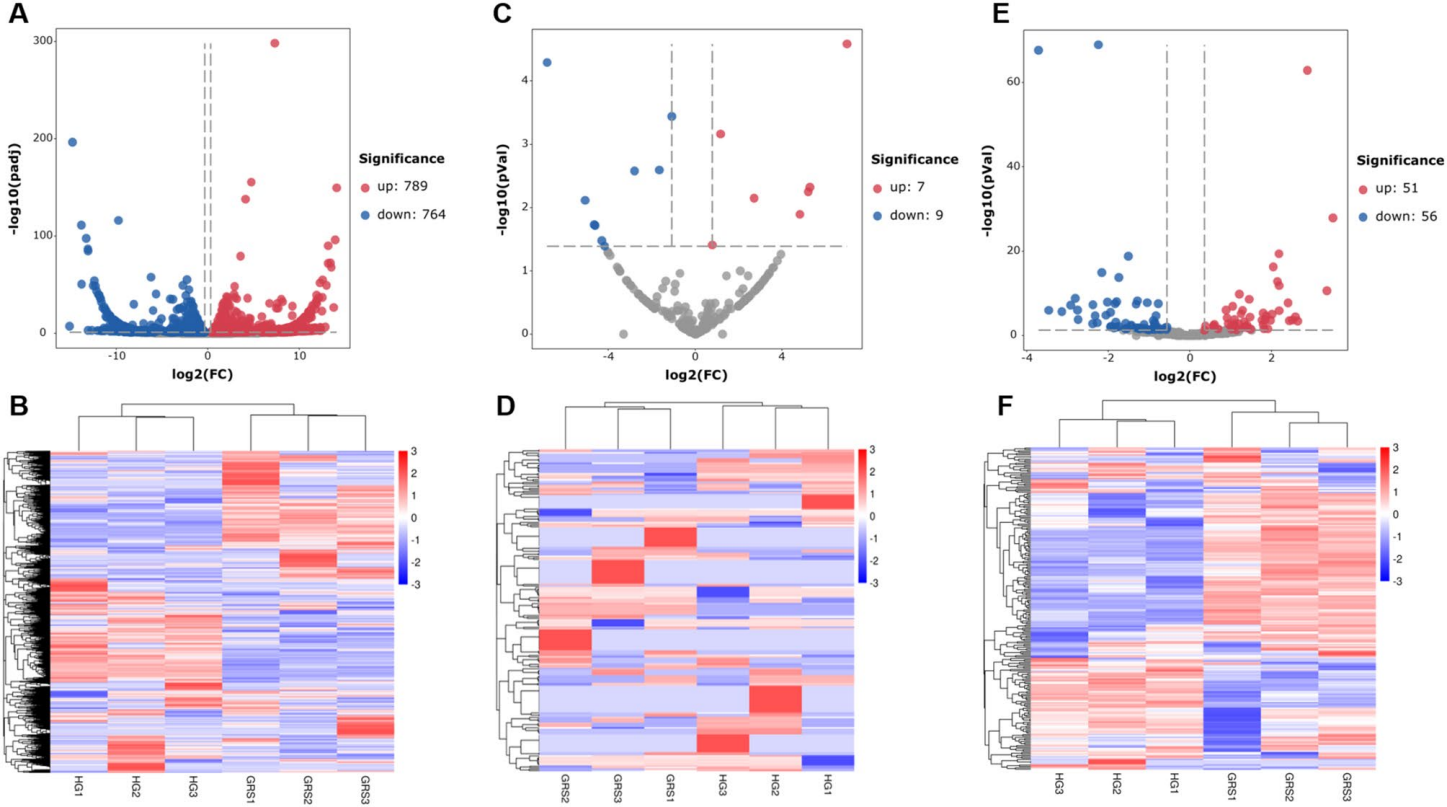
Expression profiling changes of ncRNAs in diseased tissues (the data used in heatmaps are Z-score of expressions). (**A**) Volcano plot indicating upregulated and downregulated; (**B**) heatmap of lncRNAs; (**C**) Volcano plot indicating upregulated and downregulated circRNAs; (**D**) heatmap of circRNAs; (**E**) Volcano plot indicating upregulated and downregulated miRNAs; (**F**) heatmap of miRNAs. GRS: ginseng rusty root symptom; *HG* healthy ginseng. Image generated with R 3.6.3 (https://www.R-project.org)^51^ pheatmap package (https://cran.r-project.org/web/packages/pheatmap/) and ggplot2 package (https://ggplot2.tidyverse.org).

Figure 4A,B indicates the volcano plot and clustering map of differentially expressed lncRNAs, and detailed information of differentially expressed lncRNAs is shown in Table S3. The results showed that there were 1553 differentially expressed lncRNAs (789 upregulated, 764 downregulated). Figure 4C,D indicates the volcano plot and clustering map of differentially expressed circRNAs. The results showed that there were 16 differentially expressed circRNAs. Compared with the HG group, there were seven upregulated and nine downregulated in the GRS group (Table S4). Interestingly, many circRNAs in the same group also showed significant differences in their expression levels, and some circRNAs were not expressed in a certain sample. Considering that a large number of circRNAs could not be detected in some samples, we showed the read count and RPM of differentially expressed circRNAs in each sample (Table S5). Because there were few reports about circRNAs in ginseng and it may need further study. The analysis of differentially expressed miRNAs is shown in Fig. 4E,F. The results showed that 107 genes were counted, of which 51 were upregulated and 56 were downregulated (Table S6).

### Prediction and functional analysis of differentially expressed ncRNA target genes

To investigate the potential regulatory role of ncRNAs in ginseng rusty root tissue, we further used GO and KEGG analysis to predict ncRNA target genes' function.

**Figure 5:**
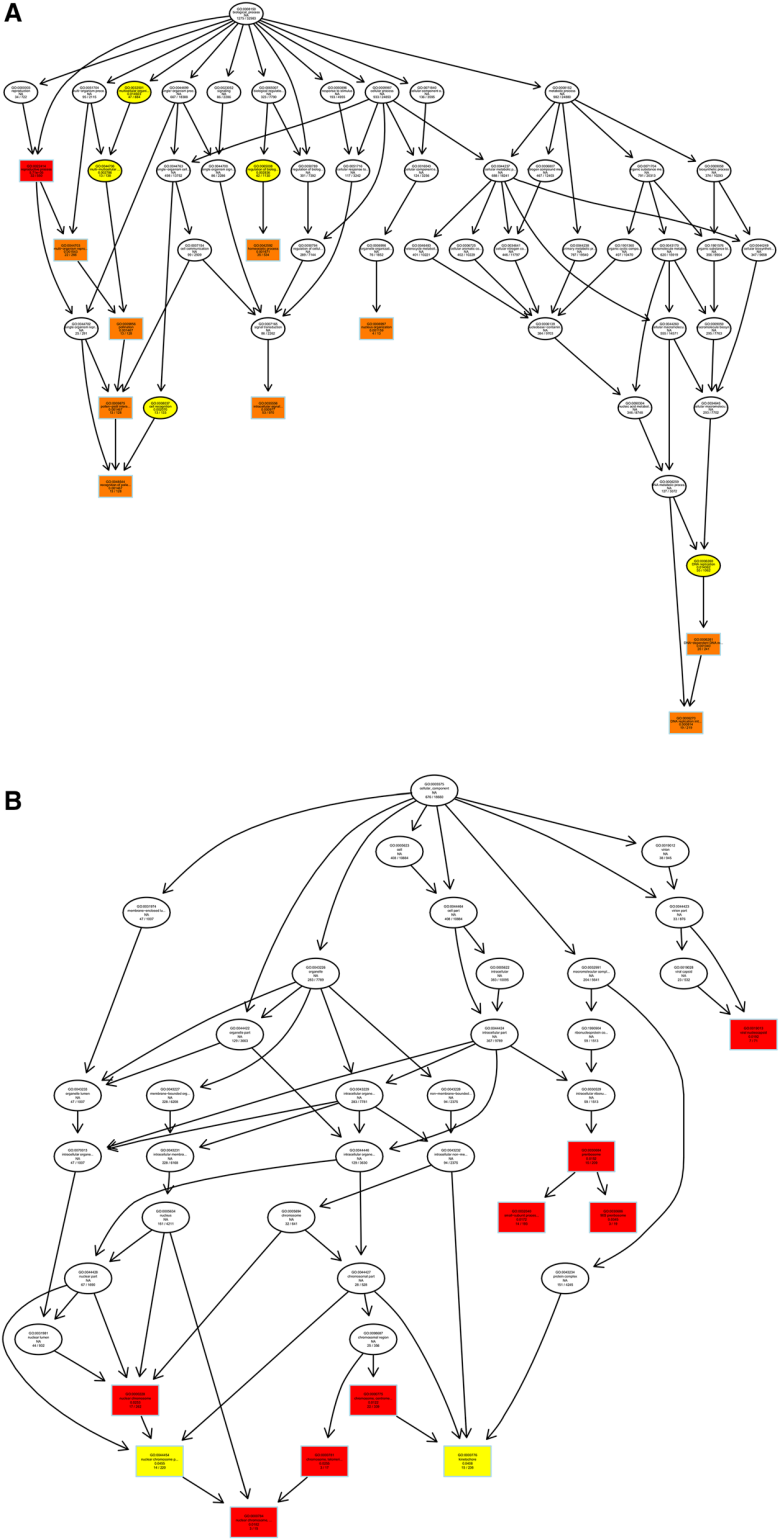

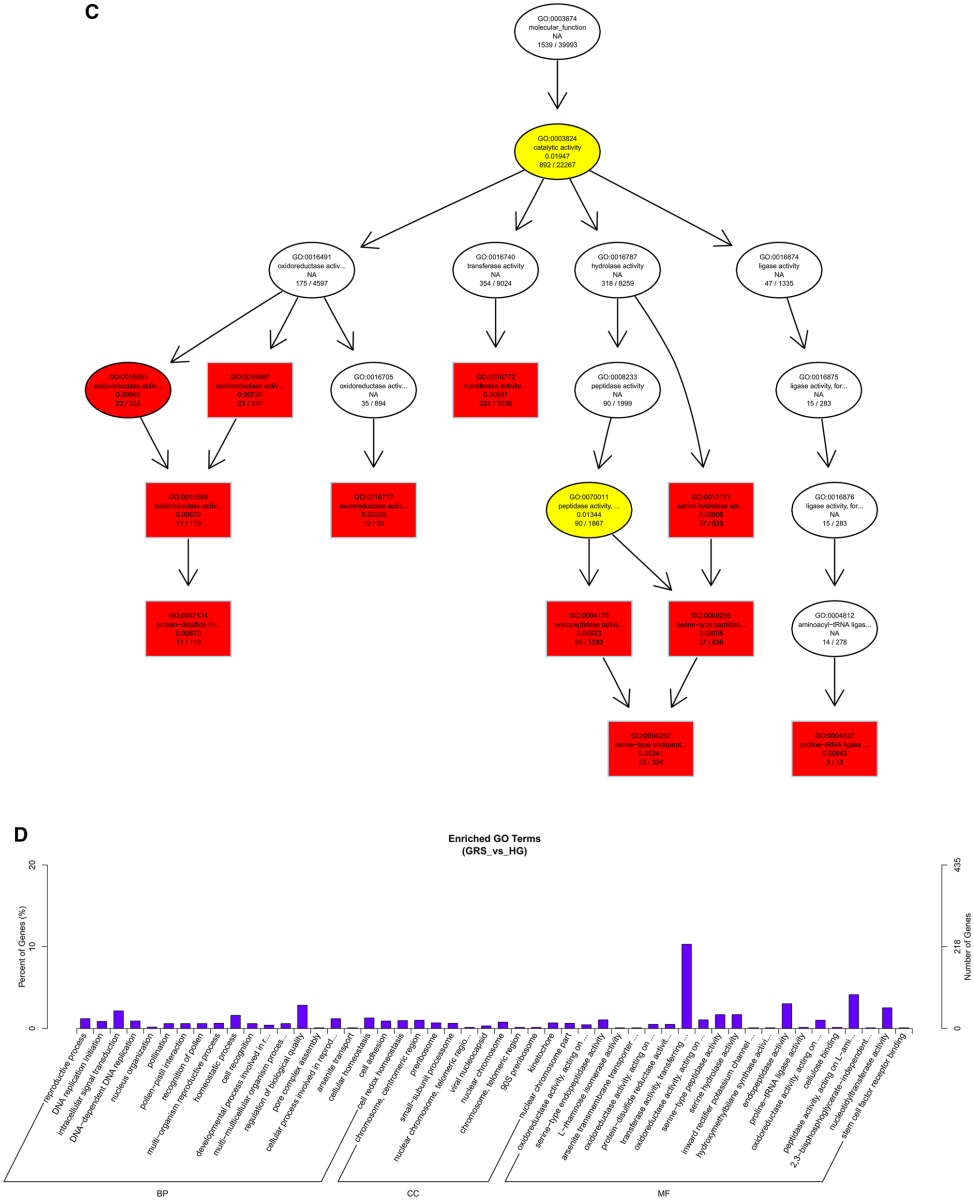
Differentially expressed lncRNAs in ginseng tissues predicted the GO enrichment analysis of target genes according to the co-location. (**A**) The directed acyclic graph of the BP part; (**B**) the directed acyclic graph of the CC part; (**C**) the directed acyclic graph of the MF part; (**D**) statistical histogram. Image generated with R 3.6.3 (https://www.R-project.org)^51^ ggplot2 package (https://ggplot2.tidyverse.org).

We predicted the target genes of lncRNAs by co-location and expression correlation between lncRNAs and protein-coding genes. Directed acyclic graphs (DAGs) and histograms of differential lncRNAs based on co-location predictions of target gene GO functional annotation analysis results are shown in Fig. 5. The biological process (BP) part is mainly enriched in intracellular signal transduction and regulation of biological quality, while the cellular component (CC) part is mainly enriched in chromosome and preribosome, the most significant molecular function (MF) part enriched for oxidoreductase activity, transferase activity, and hydrolase activity.

**Figure 6:**
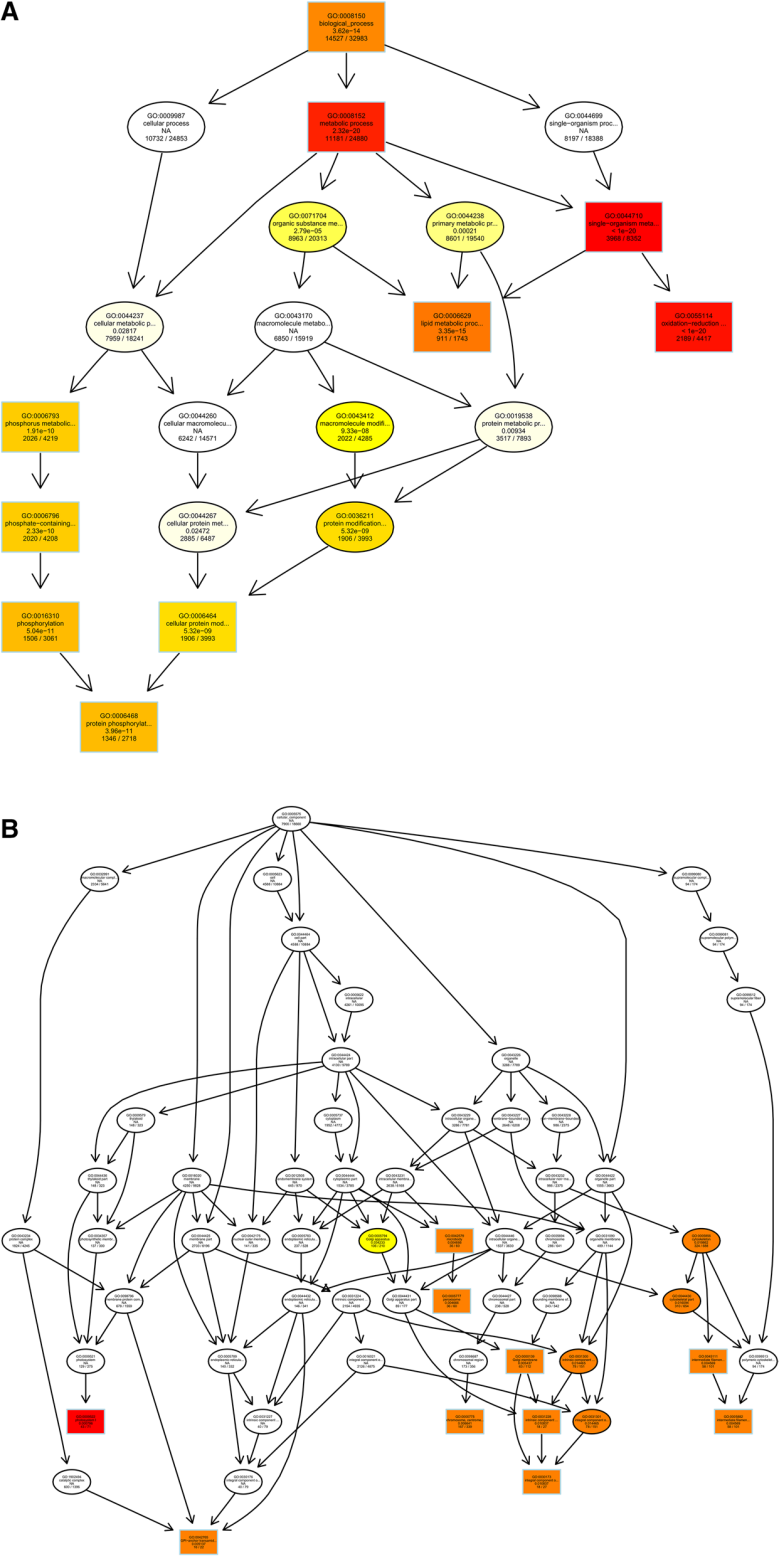

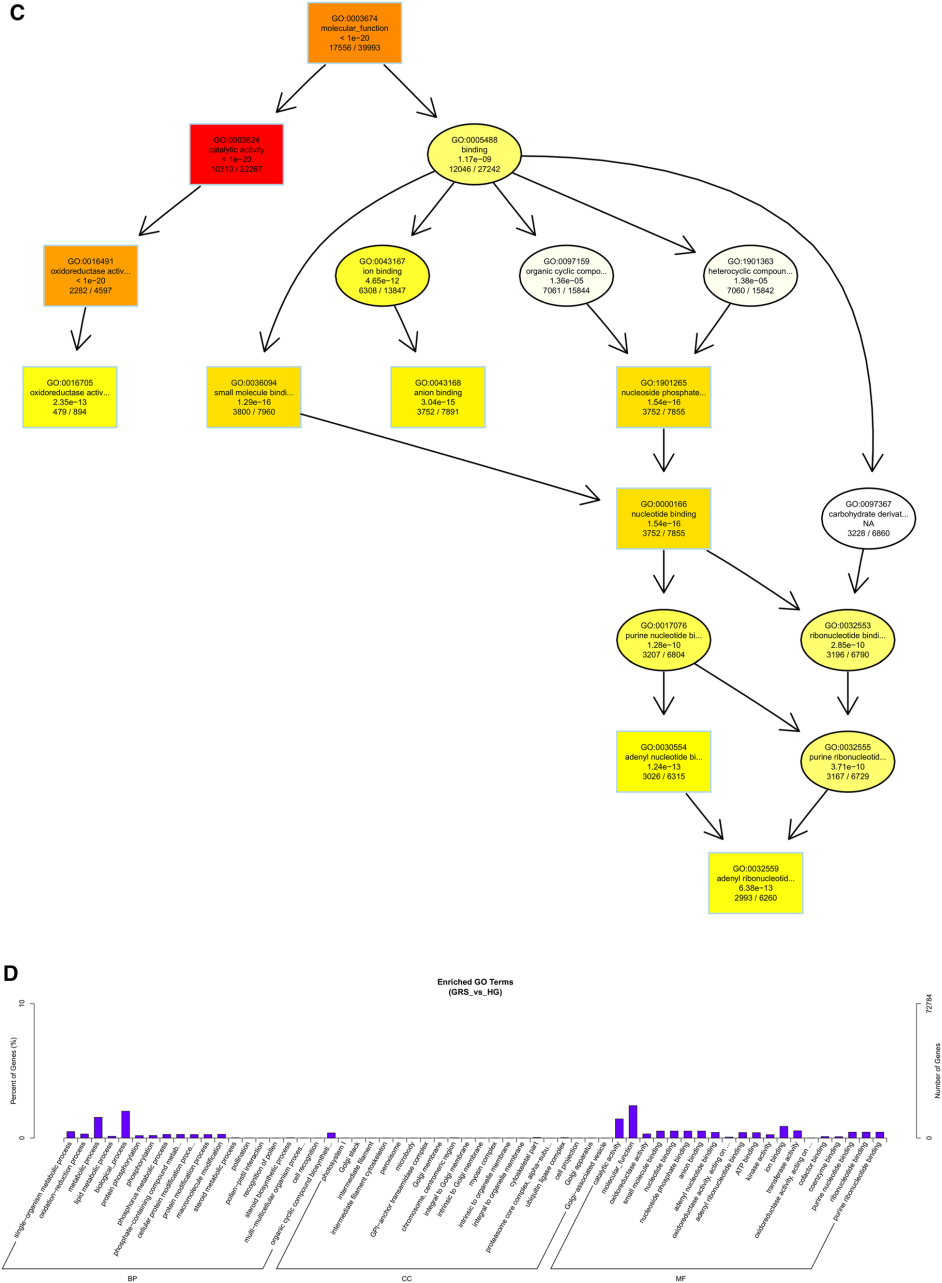
Differentially expressed lncRNAs in ginseng tissues predicted the GO enrichment analysis of target genes according to the expression correlation. (**A**) The directed acyclic graph of the BP part; (**B**) the directed acyclic graph of the CC part; (**C**) the directed acyclic graph of the MF part; (**D**) statistical histogram. Image generated with R 3.6.3 (https://www.R-project.org)^51^ ggplot2 package (https://ggplot2.tidyverse.org).

As shown in Fig. 6, we performed GO functional annotation of the target genes predicted by expression of differentially expressed lncRNAs. The BP part was significantly enriched in protein phosphorylation, lipid metabolic process, and oxidation–reduction process. The CC part is significantly enriched in photosystem I, integral component to Golgi membrane and intermediate filament. And, the MF part is mainly enriched in oxidoreductase activity and adenyl ribonucleotide binding.

**Figure 7:**
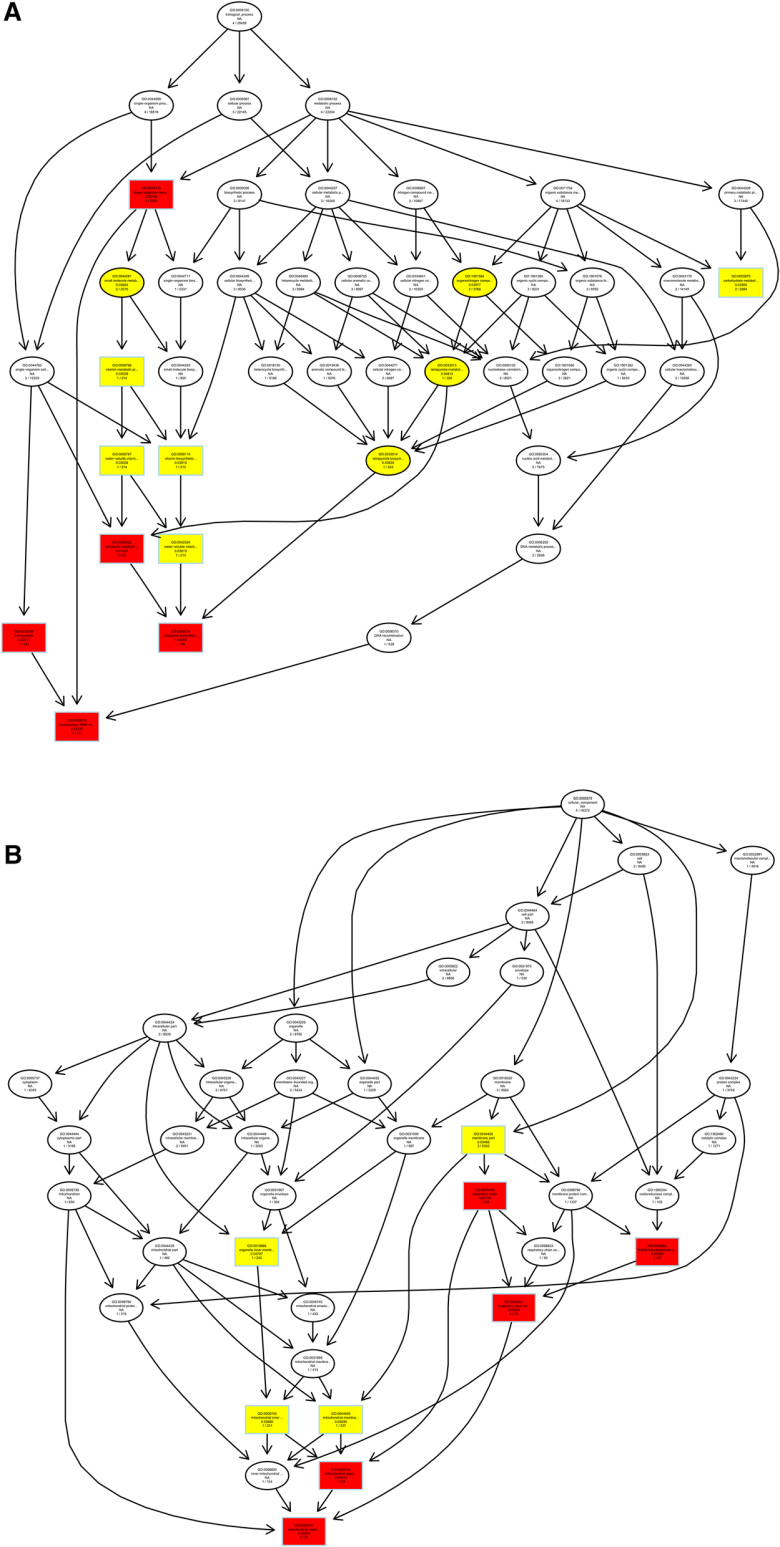

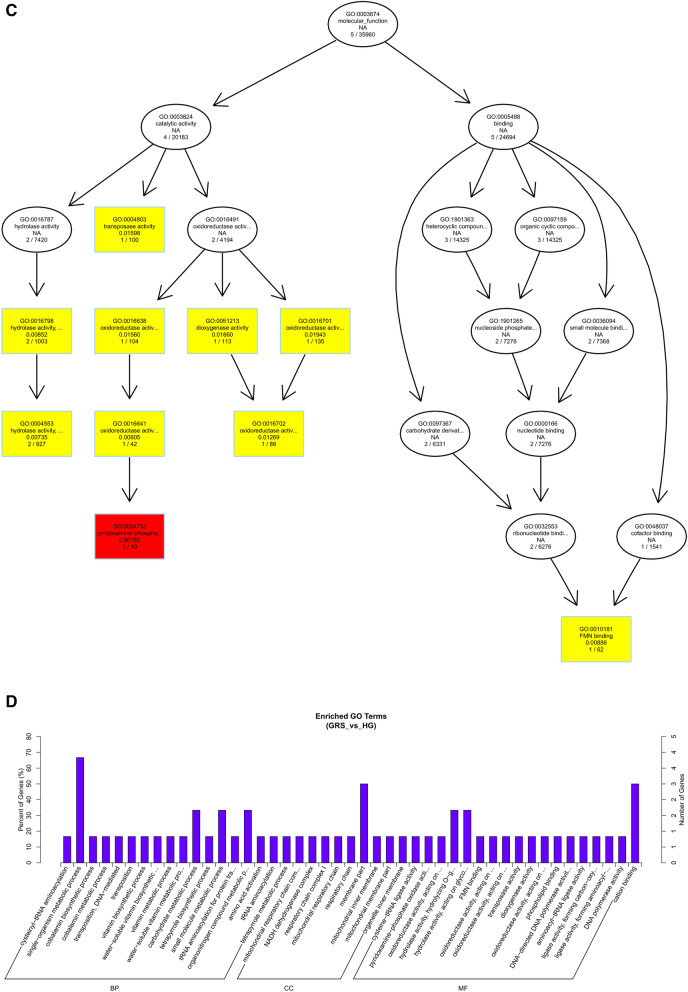
Differentially expressed circRNAs in ginseng tissues predicted the GO enrichment analysis of target genes. (**A**) The directed acyclic graph of the BP part; (**B**) the directed acyclic graph of the CC part; (**C**) the directed acyclic graph of the MF part; (**D**) statistical histogram. Image generated with R 3.6.3 (https://www.R-project.org)^51^ ggplot2 package (https://ggplot2.tidyverse.org).

After obtaining differentially expressed circRNAs, we analyzed their source genes. Through GO enrichment analysis, we found that these genes were mainly significantly enriched in the single-organism metabolic process, membrane part, and cation binding (Fig. 7).

**Figure 8:**
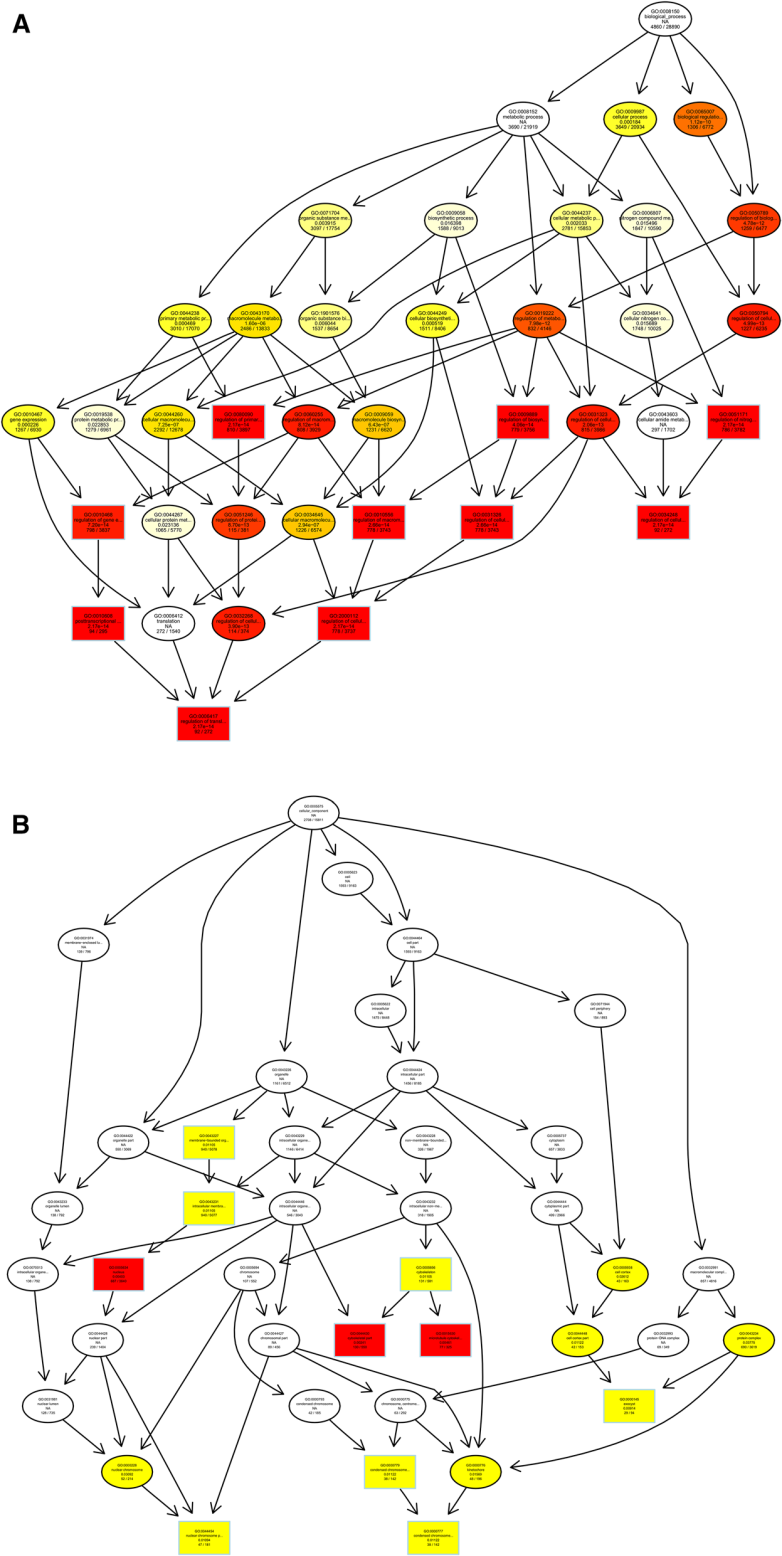

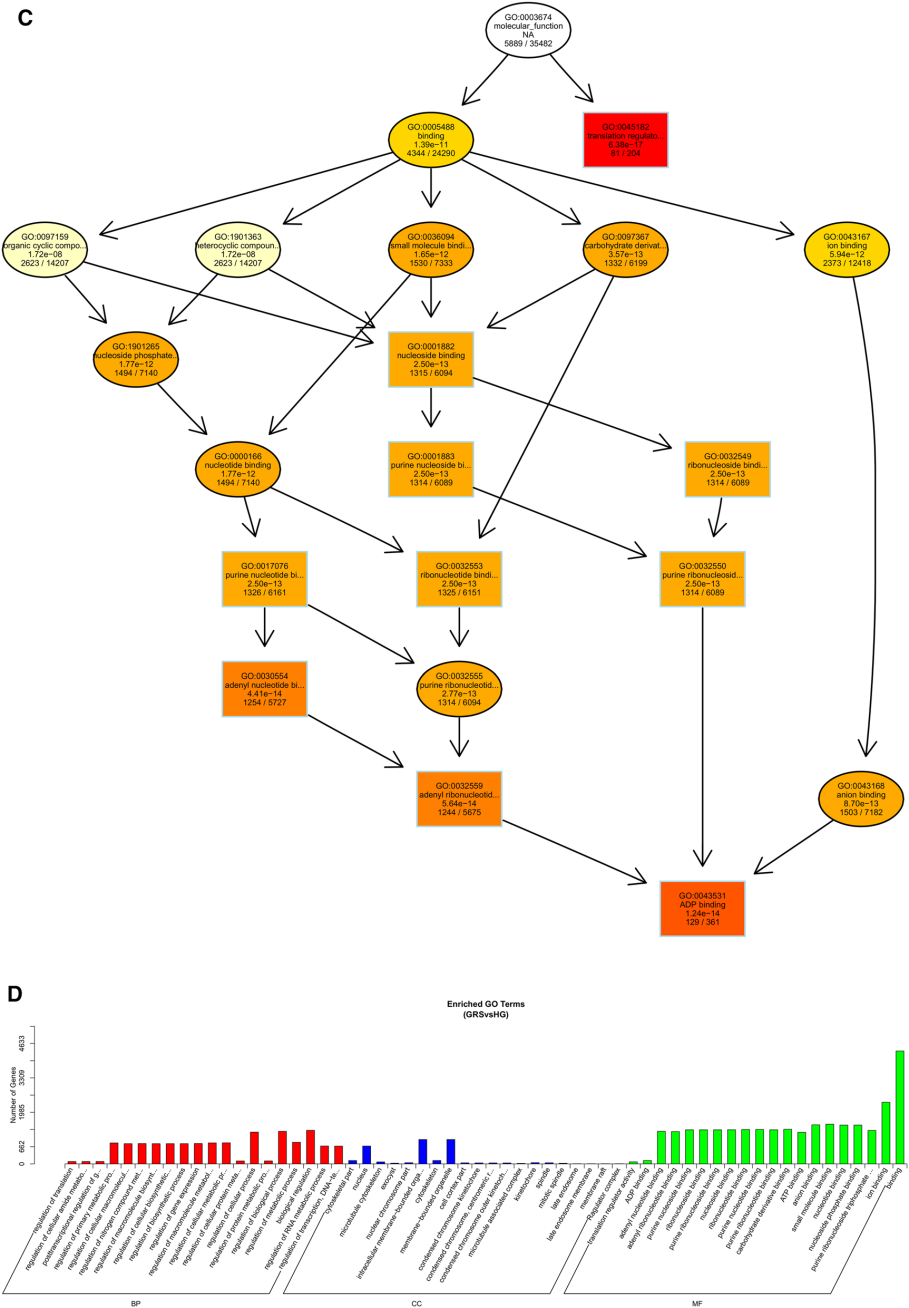
Differentially expressed miRNAs in ginseng tissues predicted the GO enrichment analysis of target genes. Image generated with R 3.6.3 (https://www.R-project.org)^51^ ggplot2 package (https://ggplot2.tidyverse.org).

Through the GO enrichment analysis of miRNA target genes, we found that in the BP part, miRNA target genes were mainly enriched in the biological regulation and regulation of the cellular process, and specifically in the regulation of translation and regulation of cellular amide metabolic process. Only the nucleus was significantly enriched in the CC part. Also, translation regulator activity and ADP binding were significantly enriched in the MF fraction, and binding enriched the most target genes (Fig. 8). This result suggests that differentially expressed miRNAs may be involved in multiple regulatory processes and cellular structure composition in ginseng tissues.

**Figure 9:**
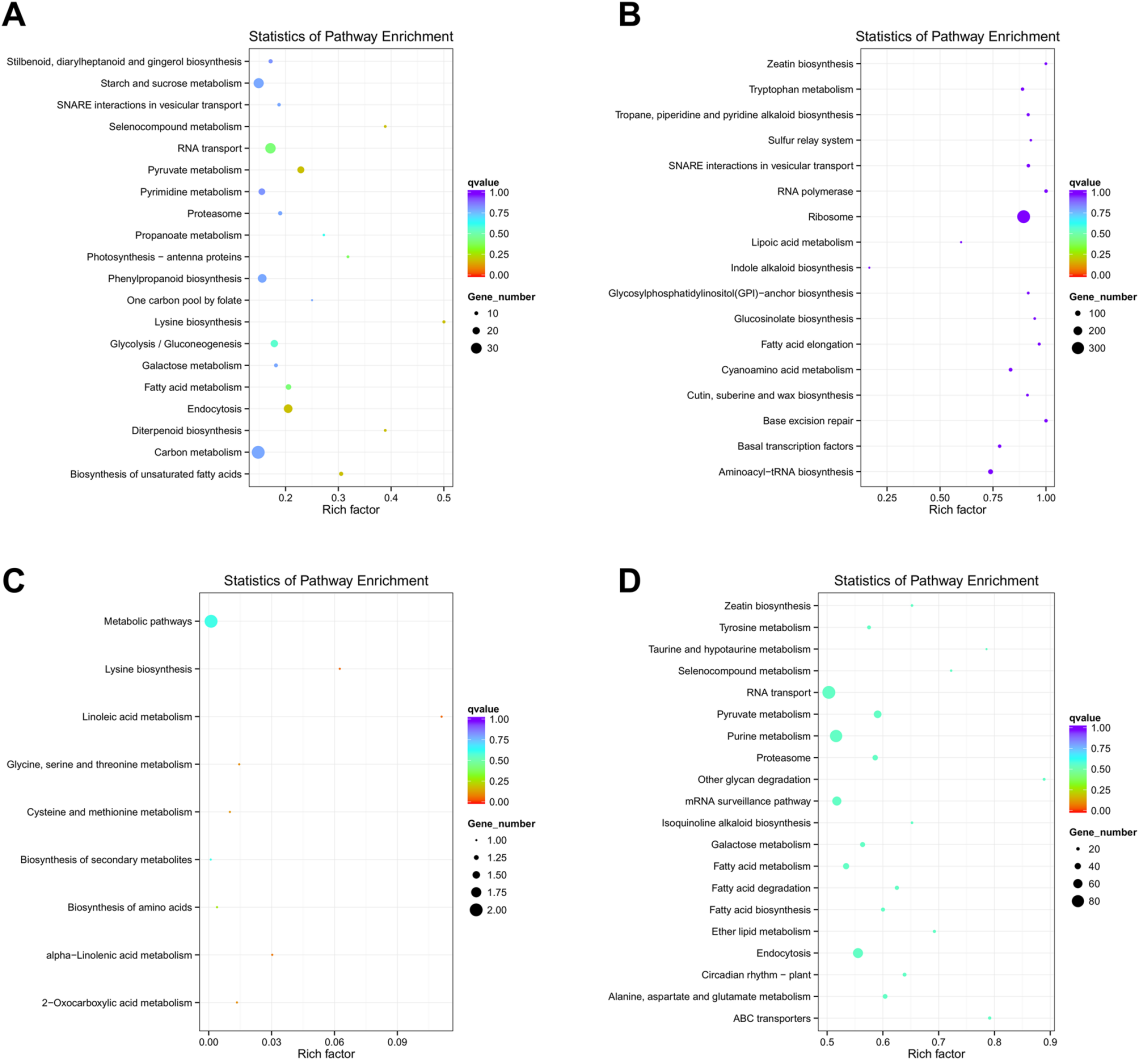
Scatter plot of KEGG pathway enrichment results for differentially expressed ncRNAs target genes. (**A**) Co-location of lncRNA; (**B**) expression correlation of lncRNA; (**C**) circRNA; (**D**) miRNA. Image generated with R 3.6.3 (https://www.R-project.org)^51^ ggplot2 package (https://ggplot2.tidyverse.org).

When the data of expression and co-location of differentially expressed lncRNA genes are used for KEGG analysis, the most significant related pathways were ribosome, aminoacyl-tRNA biosynthesis (Fig. 9A), pyruvate metabolism, and endocytosis (Fig. 9B). In the results of KEGG analysis of differentially expressed circRNAs, the most significantly involved pathways were mismatch repair, mRNA surveillance pathway, and biosynthesis of secondary metabolites (Fig. 9C). For KEGG analysis of differentially expressed miRNAs, the most significantly involved pathways were endocytosis, purine metabolism, and ABC transporters (Fig. 9D).

### Real-time quantitative polymerase chain reaction (qPCR) verified the expression of ncRNAs

**Figure 10:**
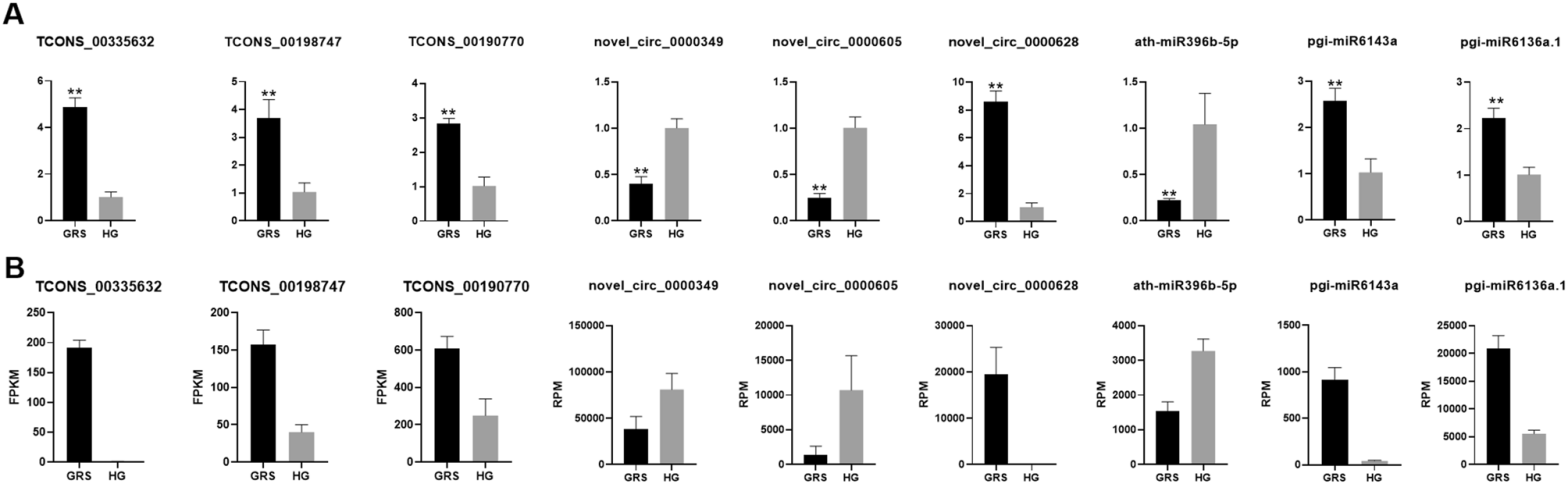
qRT-PCR validation of significant differentially expressed genes. (**A**) qRT-PCR, the data were presented as the mean ± SEM (n = 3); **p < 0.05; (**B**) RNA-seq. The figure was created by using GraphPad Prism 7 (https://www.graphpad.com/).

To verify the accuracy of RNA-seq results and provide the basis for further research, we randomly selected 9 ncRNAs (3 each for lncRNA, circRNA and miRNA) from differentially expressed ncRNAs for qRT-PCR analysis. The ncRNAs TCONS_00335632, TCONS_00198747, TCONS_00190770, novel_circ_0000349, novel_circ_0000605, novel_circ_0000628, ath-miR396b-5p, pgi-miR6143a, and pgi-miR6136a.1 were analyzed by qPCR (Fig. 10A). The expression levels of corresponding ncRNAs obtained by RNA-seq are shown in Fig. 10B. All the validation results fully proved the reliability and accuracy of the transcriptome sequencing data.

### Transcriptome association analysis

**Figure 11:**
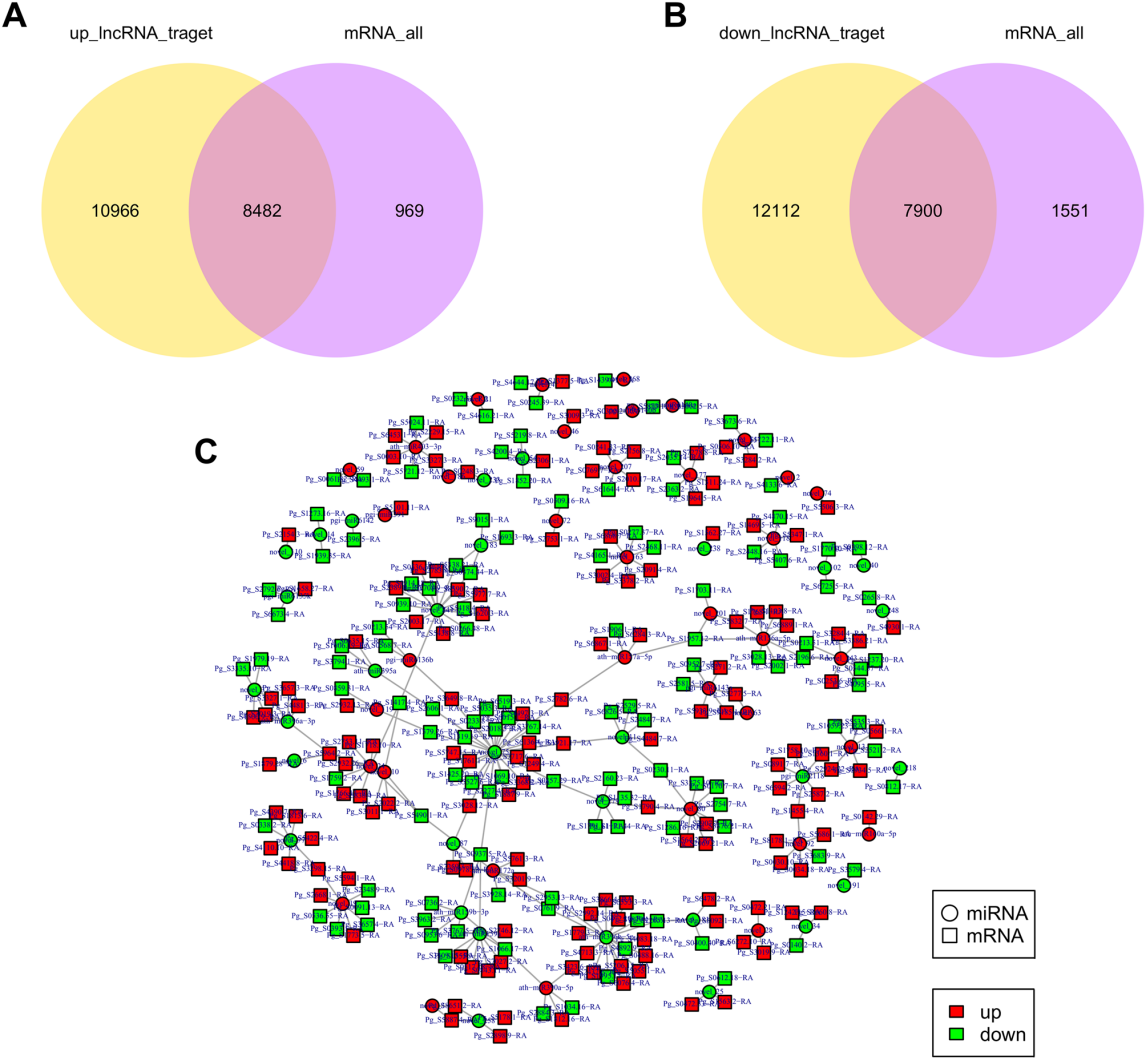
Association analysis of mRNA and ncRNA in the diseased tissues. (**A**) Venn diagram showing the overlap number of targeted mRNA of up regulated lncRNAs and differentially expressed mRNAs; (**B**) Venn diagram showing the overlap number of targeted mRNA of down regulated lncRNAs and differentially expressed mRNAs; (**C**) Interaction network of miRNA-mRNA in ginseng tissues. Image generated with R 3.6.3 (https://www.R-project.org)^51^ ggplot2 package (https://ggplot2.tidyverse.org) and network diagram made by Cytoscape sofware 3.8.2 (https://cytoscape.org/)^57^.

Aiming at the correlation analysis of lncRNAs and mRNAs, we analyzed the intersection of target genes of differentially expressed lncRNAs with differentially expressed genes. The intersection of target genes of differentially expressed lncRNAs and differentially expressed genes was shown by the Venn diagram (Fig. 11A,B), the up- and down-regulated expression of lncRNAs had 8482 and 7900 target genes overlapping with differentially expressed genes. We compared miRNAs' target genes with mRNAs and obtained 157 miRNA-mRNA pairs with potential negative regulatory relationships. As shown in Fig. 11C and Table S7, of the 85 pairs in which miRNA was down-regulated while mRNA was up-regulated, ath-miR396b-5p, pgi-miR2118, novel_241, and novel_75 had higher degrees. At the same time, we obtained 72 pairs of down-regulated miRNAs and up-regulated mRNAs, among which the degree of ath-miR156a-5p and novel_80 was greater than 5 (Fig. 11C and Table S8). These findings indicate that these miRNAs may interact with a larger number of genes, and it is speculated that these miRNAs may play an essential regulatory role in response to GRS. For example, ath-miR396b-5p potentially targets the defense-related gene NONHOST RESISTANCE1 (NHO1) (Pg_S6553.3, Pg_S0488.16), and pgi-miR2118 potentially targets the disease resistance gene ribosomal protein S2 (RPS2) (Pg_S2587.2).

**Figure 12:**
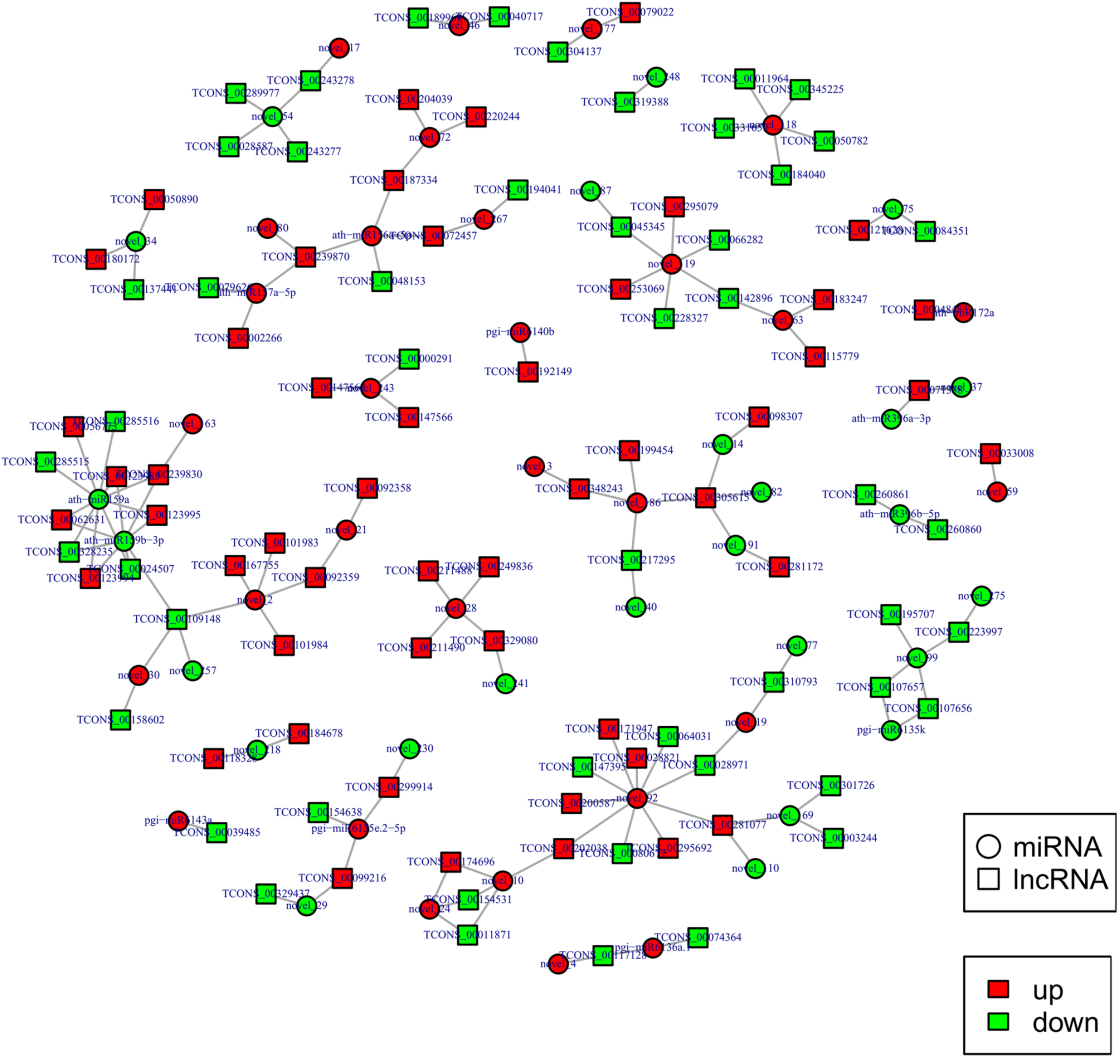
Interaction network of miRNA-lncRNA in ginseng tissues. Network diagram made by Cytoscape sofware 3.8.2 (https://cytoscape.org/)^57^.

The lncRNA can be the precursor molecule of miRNA, so we analyzed the homology of lncRNA and miRNA precursors. As shown in Table S9, we found 31 potential homologous relationships. In addition, we constructed a miRNA-lncRNA interaction network based on the targeting relationship between differentially expressed miRNAs and differentially expressed lncRNAs (Fig. 12). In the network, we found 65 potential targeting relationships (Tables S10, S11). The miRNAs with high degree are ath-miR159a, ath-miR159b-3p and novel_92.

**Figure 13:**
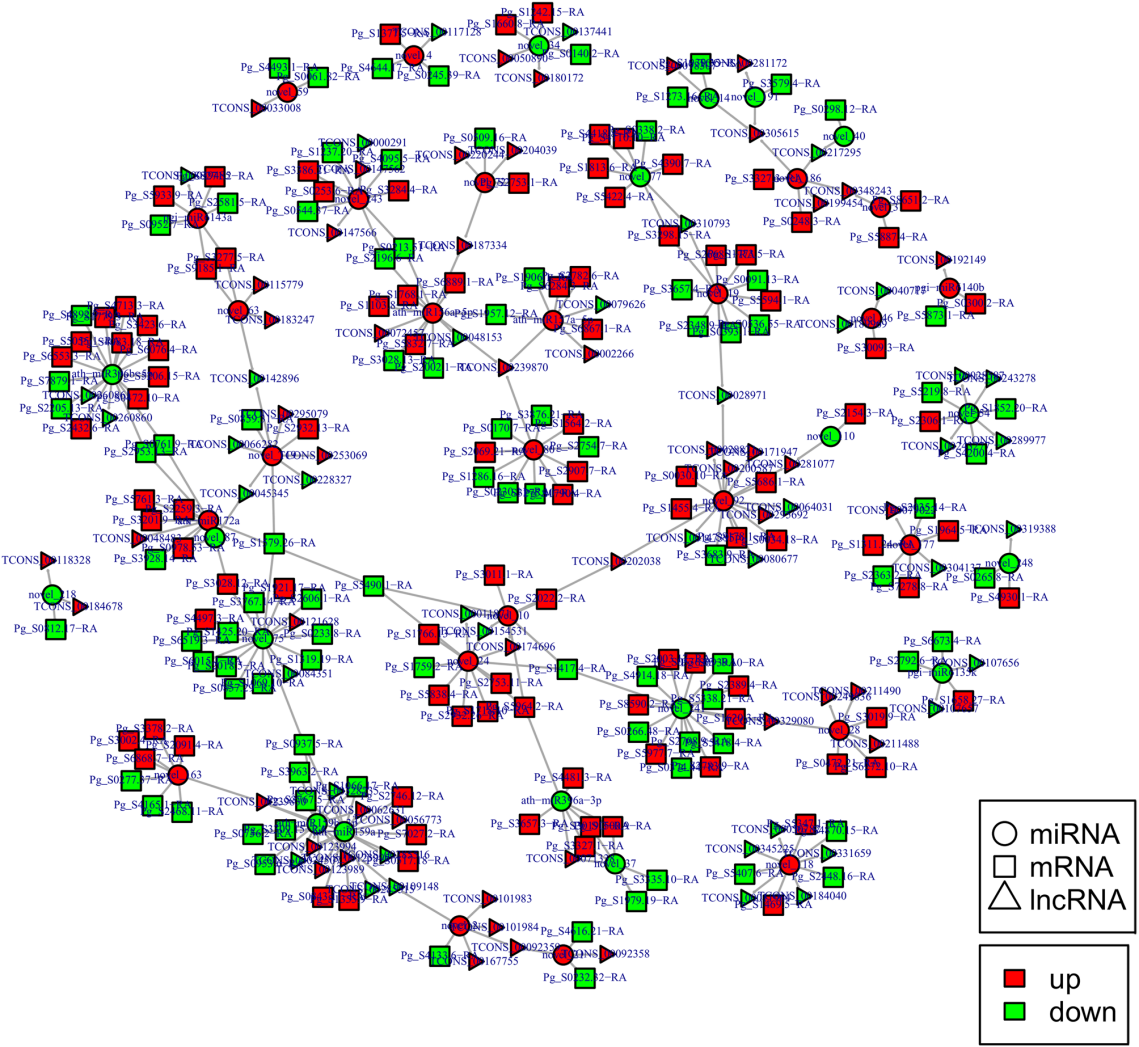
Interaction network of lncRNA-miRNA-mRNA in ginseng tissues. Network diagram made by Cytoscape sofware 3.8.2 (https://cytoscape.org/)^57^.

Based on the competing endogenous RNA (ceRNA) theory, lncRNA gene pairs with the same miRNA binding site were searched, and lncRNA gene pairs with lncRNA as a decoy, miRNA as the core, and mRNA as the target were constructed to construct the regulatory network of ceRNA (Fig. 13). A total of 90 lncRNAs, 43 miRNAs, and 191 mRNAs were found to have regulatory relationships with at least one other RNA species (Table S12). The degrees of miRNAs were generally high in the constructed interaction networks, with ath-miR396b-5p, ath-miR156a-5p, ath-miR159a, ath-miR159b-3p, novel_24, novel_243, novel_19, and novel_75 having degrees greater than 10, which were in the more central position of the interaction networks. The degrees for lncRNA and mRNA are generally 1 and 2.

## Discussion

This work investigated the differential expression of ncRNAs in GRS tissues compared to HG tissues. The potential functions of differentially expressed ncRNAs were predicted by GO and KEGG pathway analysis, and regulatory networks of mRNAs and ncRNAs were constructed. These findings suggest that ncRNAs may play a regulatory role in diseased tissues.

NcRNA is a class of RNA molecules that cannot translate proteins. According to their morphology and function, ncRNAs can be divided into various types. As the functional studies of non-coding RNAs continue to be explored, the various types of RNAs' regulatory mechanisms are gradually uncovered^24,25^. The network of interactions at the transcriptional level cannot be ignored. Currently, the mechanisms of transcriptional regulation of mRNAs and ncRNAs in GRS are unknown. Therefore, our study analyzed lncRNAs, circRNAs, and miRNAs in diseased tissues and predicted the functions and regulatory roles between mRNAs and them, which is vital for further in-depth and comprehensive studies on the mechanisms of GRS.

A total of 17,645 lncRNAs, 245 circRNAs and, 299 miRNAs were obtained by rigorous screening and prediction with a variety of bioinformatics software and online tools. Compared to previous studies, we predicted more noval ncRNAs in ginseng, so the characteristics of ncRNAs in the samples were described as necessary. Then, we conducted differential expression analysis and cluster analysis of ncRNAs in GRS tissues and HG tissues. The results showed 1553 lncRNAs, 16 circRNAs and, 107 miRNAs significantly differently expressed in GRS tissue. Interestingly, due to their circular structure, circRNAs are generally highly stable and conserved compared to other RNA types. However, we also found large differences in circRNAs in the same set of samples (Fig. 4D). Given the relatively few reports on circRNAs in ginseng, the reasons for this may require further investigation.

To understand the biological functions and potential regulatory mechanisms of ncRNAs in GRS tissues, we predicted the screened differentially expressed ncRNAs' target genes and then performed GO and KEGG functional enrichment analysis on the obtained target genes. The mechanisms by which lncRNAs regulate target genes are diverse, and we predict the differential expression of lncRNA target genes by the most common ways of acting downstream (co-location and expression). Co-location means that the lncRNA may have a regulatory effect on a nearby protein-coding gene; expression means that the lncRNA regulates downstream genes through expression correlation. In the GO enrichment analysis of target genes with differential lncRNAs screened by co-location, we found that the target genes were significantly enriched in " regulation of biological quality" (GO:0065008) and specifically in " homeostatic process" (GO:0042592). These suggest that differentially expressed lncRNAs in ginseng diseased tissues may be involved in regulating the maintenance of internal homeostasis. Interestingly, we also found enrichment for the "reproductive process"(GO:0022414) in this section, whereas no reports of ginseng reproduction affected by GRS have been reported so far. The enrichment of chromosomal, ribosome-associated terms (GO:0000228, GO:0000775, GO:0030684, etc.) suggests that lncRNAs may be involved in transcriptional regulation translation in ginseng cells. Besides, lncRNAs may also be involved in regulating various enzyme activities, including oxidoreductase and transferase, in ginseng tissue (GO:0003674). The results of GO enrichment analysis of differentially expressed miRNAs showed that, compared to healthy ginseng, differentially expressed miRNAs in red skin tissues exhibited differential enrichment for the regulation of translation (GO:0006417, GO:0045182), regulation of transcription (GO:0006355), and "nucleotide binding" (GO:0000166).

In the results of the KEGG enrichment analysis of target genes that differentially expressed ncRNAs, we can understand that ncRNA may be involved in the synthesis of various amino acids and the ribosome pathway. This result may indicate that ncRNAs mainly regulate the translation process of diseased tissue. Moreover, both differentially expressed lncRNAs and miRNAs are involved in the fatty acid synthesis, metabolism, or elongation under this disease's background. Fatty acids are crucial components of cellular membranes, suberin, and cutin waxes that provide structural barriers to the environment. On the one hand, cutin and wax can physically shield against pathogenic bacteria by influencing the physical properties (mechanical strength and hydrophobicity) of the stratum corneum; on the other hand, they can directly control the proliferation and differentiation of pathogenic bacteria through hydroxy fatty acids and other bacteriostatic components to achieve their chemical defense function^26–28^. The “cutin, suberine and wax biosynthesis” pathway was also enriched in the expression of differentially expressed lncRNAs. This result may mean that ncRNA's regulatory effect on fatty acids is significant in GRS^29^. Alkaloids are of great significance for plants to resist pathogens and play a role of poison or expulsion^30^. In our enrichment results, ncRNAs are involved in regulating a variety of alkaloid biosynthesis, indicating that ncRNAs may play a significant role in phytochemical resistance in ginseng diseased tissues.

In our analysis, 25 known miRNAs were differentially expressed (Table S6). For example, miR6143a have been reported to have a potential role in callus development and maintenance^19^. Moreover, mi6135k, miR6140d, miR6139, miR6140a, and miR6136b are responsive to heat treatment. MiR6139 are probably involved in the homeostasis of metal ion^31^. The target gene prediction of miR2118 also indicated that miR2118 may have a regulatory effect on disease-resistance protein genes^19^. We have obtained many differentially expressed ncRNAs in GRS, but most of them are novel ncRNAs, and there are few studies on their roles. Therefore, their potential roles still need to be further explored.

To further understand the interactions between mRNAs and ncRNAs, we analyzed the correlations between differentially expressed mRNAs and ncRNAs in HG tissues and GRS tissues, and based on these data. We found substantial overlap after matching the target genes of differentially expressed lncRNAs with differentially expressed mRNAs, suggesting that many differentially expressed genes in GRS are likely to be regulated by lncRNAs.

We compared the target genes of differentially expressed miRNAs with mRNAs and constructed miRNA-mRNA networks by retaining only the negatively regulated miRNA-mRNA pairs. A total of 157 negatively regulated combinations were obtained. We found ath-miR156a-5p, novel_80, and several other miRNAs in the more central position of the network, which was hypothesized to play essential roles in GRS formation.

Considering that miRNA-lncRNA interactions may play an important regulatory role in plant response to stress^32^, we analyzed the targeting relationship between differentially expressed miRNAs and differentially expressed lncRNAs, and constructed an interaction network. It's worth noting that, we found that known miRNAs, ath-miR159a and ath-miR159b-3p, have a high degree in the network. The miR159 family can respond to various environmental stresses^33^, suggesting that ath-miR159a and ath-miR159b-3p may play an important role in GRS through targeted regulation of lncRNAs.

Finally, we constructed an mRNA-lncRNA-miRNA interaction network based on the ceRNA mechanism^34,35^. Some lncRNAs and mRNAs were found to be regulated by the same miRNA, which may indicate that these RNAs may be involved in the relevant regulation in GRS through the ceRNA mechanism.

In summary, we identified lncRNAs, circRNAs, and miRNAs in GRS tissues and HG tissues and performed functional enrichment analysis of the screened differentially expressed ncRNAs. We obtained 17,645 lncRNAs, 245 circRNAs, and 299 miRNAs from the ginseng root tissues and characterized the obtained ncRNAs. By GO and KEGG analysis, we found that lncRNAs may be involved in homeostasis regulation, and lncRNAs, circRNAs, and miRNAs are involved in fatty acid-related regulation that alterations in fatty acid-related pathways may play a key role in GRS. Differentially expressed ncRNAs play an essential role in transcription and translation, primary metabolism, and secondary metabolism in ginseng tissues. Finally, we integrated the correlations between ncRNAs, constructed the corresponding interaction network, and identified ncRNAs that may play a key role in GRS. These results provide the basis for revealing the molecular mechanism of GRS and enrich our understanding of ncRNA in ginseng.

## Materials and methods

### Site description and sample collection

In previous investigations^36^, we found that the ginseng grown at a ginseng farm showed more rust root symptoms in Hunchun city (42.86′ N and 130.37′ E) in northeast China. And this farm is the first to grow ginseng; the application of pesticides strictly complies with the “Ginseng safe production technical specification of pesticide application (DB22/T 1233-2019)”.

The diseased and healthy tissues were cut from GRS and HG, and all ginseng was taken from 5-year-old ginseng. Three independent biological replicates were prepared, and each replicate included root materials from three or more ginseng plants. The permissions were obtained from concerned authorities for collection and use of sample. And all methods were performed in accordance with the relevant regulations.

### RNA extraction and quality control

RNA extraction and quality control refer to previously methods^37^. TRIzol reagent (Invitrogen, Carlsbad, CA) was used to isolate the total RNA of each sample. RNA degradation and contamination were monitored on 1% agarose gels. RNA purity was checked using the NanoPhotometer spectrophotometer (IMPLEN, CA, USA). RNA concentration was measured using Qubit RNA Assay Kit in Qubit 2.0 Flurometer (Life Technologies, CA, USA). RNA integrity was assessed using the RNA Nano 6000 Assay Kit of the Bioanalyzer 2100 system (Agilent Technologies, CA, USA).

### Library preparation for RNA sequencing

A total of six complementary DNA (cDNA) libraries for screening lncRNA and circRNA, three GRS, and three HG were constructed in this part. A total amount of 3 µg RNA per sample was used as input material for RNA sample preparation. Sequencing libraries were generated using NEBNext Ultra RNA Library Prep Kit for Illumina (NEB, USA), following the manufacturer’s recommendations, and index codes were added to attribute sequences to each sample^38^. Briefly, mRNA was purified from the total RNA using poly-T oligo-attached magnetic beads. Fragmentation was conducted using divalent cations under elevated temperatures in NEBNext First Strand Synthesis Reaction Buffer (5X). The first strand cDNA was synthesized using a random hexamer primer and M-MuLV Reverse Transcriptase (RNase H-). The second strand cDNA synthesis was subsequently performed using DNA Polymerase I and RNase H. Remaining overhangs were converted into blunt ends via exonuclease/polymerase activities. After adenylation of 3′ ends of DNA fragments, NEBNext Adaptor with a hairpin loop structure was ligated to prepare for hybridization. To select cDNA fragments of preferentially 150–200 bp in length, the library fragments were purified with the AMPure XP system (Beckman Coulter, Beverly, USA). Then, 3 µl USER Enzyme (NEB, USA) was used with size-selected, adaptor-ligated cDNA at 37 °C for 15 min, followed by 5 min at 95 °C before PCR. PCR was performed with Phusion High-Fidelity DNA polymerase, Universal PCR primers, and Index (X) Primer.

We also constructed six small RNA libraries, three GRS, and three HG. A total amount of 3 µg total RNA per sample was used as input material for the small RNA library. Sequencing libraries were generated using NEBNext Multiplex Small RNA Library Prep Set for Illumina (NEB, USA), following the manufacturer's recommendations, and index codes were added to attribute sequences to each sample^39^. Briefly, the NEB 3′ SR Adaptor was directly and specifically ligated to the 3′ end of miRNA, siRNA, and piRNA. After the 3′ ligation reaction, the SR RT Primer hybridized to the excess of 3′ SR Adaptor (that remained free after the 3′ ligation reaction). It transformed the single-stranded DNA adaptor into a double-stranded DNA molecule. This step is important to prevent adaptor-dimer formation; additionally, dsDNA is not substrates for ligation-mediated by T4 RNA Ligase 1. Therefore, do not ligate to the 5′ SR Adaptor in the subsequent ligation step. Adapter 5′ ends were ligated to 5′ ends of miRNAs, siRNAs, and piRNAs. Then, the first strand cDNA was synthesized using M-MuLV Reverse Transcriptase (RNase H-). PCR amplification was performed using LongAmp Taq 2X Master Mix, SR Primer for Illumina, and index (X) primer. PCR products were purified on an 8% polyacrylamide gel (100 V, 80 min). DNA fragments corresponding to 140–160 bp (the length of small noncoding RNA plus the 3′ and 5′ adaptors) were recovered and dissolved in an 8 µl elution buffer.

In the end, all library quality was assessed on the Agilent Bioanalyzer 2100 system.

### Clustering and sequencing

Clustering of the index-coded samples was performed on a cBot Cluster Generation System using TruSeq PE Cluster Kit v3-cBot-HS (Illumia), according to the manufacturer’s instructions^40^. After cluster generation, cDNA library preparations were sequenced on an Illumina Hiseq PE150 platform and, 150 bp paired-end reads were generated. The small library preparations were sequenced on an Illumina Hiseq SE50 platform, and 50 bp paired-end reads were generated. The raw RNA-seq data are freely available in the NCBI database under accession no. PRJNA684799 and PRJNA684889.

### Data analysis

#### Quality control of sequencing data

Raw data (raw reads) of the fastq format were first processed through in-house Perl scripts. In this step, clean data were obtained by removing reads containing the adapter, reads containing poly-N, and low-quality reads from raw data. At the same time, Q20, Q30, and GC content of the clean data were calculated. Clean reads obtained were used for subsequent analysis. The 18-35nt range was selected from small libraries clean reads for subsequent miRNA analysis.

#### Reads mapping to the reference genome

The reference genome (http://ginsengdb.snu.ac.kr/download.php?filename=ginseng_v1.fasta) and gene model annotation files (http://ginsengdb.snu.ac.kr/download.php?filename=IPGA_v1_Jbrowse_update_1.1.gff, ginseng) were downloaded from the genome website directly^41^. The reference genome index was built using STAR, and paired-end clean reads were aligned to the reference genome using STAR (v2.5.1b). STAR used the method of the maximal mappable prefix (MMP), which can generate a precise mapping result for junction reads. The miRNA tags were mapped to Bowtie's reference sequence without mismatch to analyze their expression and distribution on the reference.

#### NcRNA identification and target gene prediction

For lncRNA, based on lncRNA's characteristics, we used a strict screening method to identify lncRNAs^42^. First, the transcript with the number of exons ≥ 2 and the length greater than 200 bp was selected. Then, Cuffcompare software was used to filter out the transcript that overlapped with the exon region of the database annotation. Thirdly, after filtering out known transcripts from the database using Cuffcompare, CPC2^43^, Pfam^44^, and CNCI^45^ software were used to screen for coding potential, and the intersection of transcripts with no coding potential from these software analyses was used as a candidate novel lncRNA dataset for this analysis. Finally, the candidate novel lncRNAs were screened and named regarding the HGNC (The HUGO Gene Nomenclature Committee) naming guidelines for lncRNAs to obtain the novel lncRNAs for this analysis.

The target genes of the lncRNAs were predicted by co-location and expression between the lncRNAs and the protein-coding genes. The screening range for target gene analysis by co-location was within 100 kb. The screening condition for prediction of lncRNA target genes by expression was a Pearson correlation coefficient greater than 0.95 and less than -0.95.

For circRNA, two most commonly used circRNA identification software (find circ and CIRI) are used for circRNA identification to improve the accuracy of circRNA identification^46,47^. And then, we predicted the potential function of circRNAs based on the corresponding relationship between circRNAs and their source genes.

For miRNAs, after obtaining clean reads of each sample, small RNAs (sRNAs) in the 15-30nt length range were screened. The length-filtered sRNAs were localized to a reference sequence using bowtie (v.1.2.2) software, and the distribution of sRNAs on the reference sequence was analyzed by comparing the specified range sequences in the miRBase database (v.21), and known miRNAs were obtained. Besides, miREvo (v.1.2) and mirdeep2 (v.0.1.2) software were integrated for novel miRNA analysis and prediction of novel miRNAs in the samples^48,49^. Family analysis was performed on the detected miRNAs to explore the occurrence of miRNA families in other species. The known miRNA used miFam.dat (http://www.mirbase.org/ftp.shtml) to look for families; novel miRNA precursor was submitted to Rfam (http://rfam.sanger.ac.uk/search/) to look for Rfam families. Finally, predicting the target gene of miRNA was performed by psRobot_tar in psRobot (v.1.2) software^50^.

#### Quantification of gene expression level

HTSeq (v.0.6.0)^51^ was used to count the read numbers mapped to each gene. FPKM of each gene was calculated based on the length of the gene and read count mapped to this gene. FPKM, the expected number of fragments per kilobase of transcript sequence per million base pairs sequenced, considers the effect of sequencing depth and gene length for the read count at the same time and is currently the most commonly used method for estimating gene expression levels.

The circRNA and miRNA expression levels were estimated by RPM (reads per million) through the following criteria: normalized expression = mapped reads count/total mapped reads*1,000,000.

#### Differential expression analysis

The R software (v.3.6.3) (https://www.R-project.org) DESeq2 package (http://www.bioconductor.org/packages/release/bioc/html/DESeq2.html) (v.1.12.0) was used to perform differential expression analysis between the two groups based on reads count^52,53^. DESeq2 provided statistical routines for determining differential expression in digital gene expression data using a negative binomial distribution model. The resulting P-values were adjusted using Benjamini and Hochberg’s approach for controlling the false discovery rate.

#### GO and KEGG enrichment analysis

GO (http://www.geneontology.org/) enrichment analysis of differentially expressed genes was implemented by the GOseq based Wallenius non-central hypergeometric distribution, in which gene length bias was corrected^54^. GO terms with corrected P-values less than 0.05 were considered significantly enriched by differential expressed genes. KEGG is a database resource for understanding high-level functions and utilities of the biological system, such as the cell, organism, and ecosystem, from molecular-level information, especially large-scale molecular datasets generated by genome sequencing and other high-throughput experimental technologies (http://www.genome.jp/kegg/). We performed KEGG enrichment analysis on differentially expressed genes using KOBAS (v.2.0) software^55^.

### Quantitative real-time RT-PCR

For lncRNA and circRNA, total RNA isolation and cDNA preparation from different tissue samples of GRS and HG were the same as those above in “RNA isolation and quality control.” The quantitative real-time PCR (qRT-PCR) analysis was performed using 2 × RealStar Green Fast Mixture (GenStar, China) and the Real-Time PCR System (Lightcycler 96, Roche, Switzerland). β-actin was used as an internal control gene. For each reaction, 0.5 μl of the forward and reverse primers and 2 μl of cDNA template were added.

In PCR validation of miRNA expression, total RNA reverse transcription was performed using the Mir-X miRNA First-Strand Synthesis kit (TaKaRa, Dalian, China). The 5′ forward primers for qRT-PCR validation of miRNAs included the entire sequence of the mature miRNAs, as suggested by the manufacturer, and the 3′primer for qRT-PCR was supplied with the kit. The qRT-PCR analysis was performed using TB Green Premix Ex Taq II (TaKaRa, Dalian, China) and the Real-Time PCR System (Lightcycler 96, Roche, Switzerland). U6 was used as the internal control. For each reaction, 0.8 μl of the forward and reverse primers and 2 μl of cDNA template were added.

All of the primers used in this study are listed in Table S13. The relative gene expression level was calculated according to the 2^−ΔΔCt^ method^56^.

### Whole transcriptome association analysis

We carried on the association analysis to the obtained data to reveal the role and interactions between differentially expressed ncRNAs and their relationship to differentially expressed genes^57^ in GRS:

1. We focused on the differentially expressed lncRNA to find the mRNA with the target relationship with the differentially expressed lncRNA. Then we crossed these mRNA with the corresponding combination of the differentially expressed mRNA to get the differentially expressed mRNA targeted by the differentially expressed lncRNA.
2. Taking the differentially expressed miRNA as the center, we searched for the mRNA that had a target relationship with the differentially expressed miRNA and then crossed these mRNAs with the corresponding combination of the differentially expressed mRNA to get the differentially expressed mRNA targeted by the differentially expressed miRNA.
3. In order to analyze the potential interactions of lncRNA-miRNA, lncRNA-miRNA pairs were found based on the homology between lncRNA and miRNA precursors. Then the miRNA-targeted lncRNAs were predicted by psRobot software, and the miRNA-lncRNA interaction network was constructed according to the obtained prediction information.
4. miRNA was used as the center of the map to associate lncRNA and mRNA. The interaction network was built based on the screening of lncRNA-miRNA-gene pairs with psRobot software.

The visualization of the above interaction network was completed by Cytoscape (v.3.8.2) software^58^.

## Supplementary Information

Supplementary Information 1.

Supplementary Information 2.

Supplementary Information 3.

Supplementary Information 4.

Supplementary Information 5.

Supplementary Information 6.

Supplementary Information 7.
